# The role of climatic variables on nest evolution in tanagers

**DOI:** 10.1002/ece3.11168

**Published:** 2024-04-01

**Authors:** Silvia Colombo, Kevin D. Newman, Naomi E. Langmore, Claire J. Taylor, Iliana Medina

**Affiliations:** ^1^ School of Biosciences University of Melbourne Parkville, Melbourne Victoria Australia; ^2^ School of Agriculture, Food and Ecosystem Sciences University of Melbourne Parkville, Melbourne Victoria Australia; ^3^ Division of Ecology and Evolution, Research School of Biology Australian National University Canberra Australian Capital Territory Australia

**Keywords:** avian nest, climatic variables, evolution, nest architecture, phylogenetic tree, tanagers

## Abstract

Avian nests are fundamental structures in avian reproduction and face strong selective forces. Climatic conditions are likely to have shaped the evolution of specific nest traits, but evidence is scarce at a macroevolutionary level. The Thraupidae family (commonly known as tanagers) is an ideal clade to understand the link between nest architecture and climate because it presents wide variation in nest traits. To understand whether climatic variables have played a role in the diversification of nest traits among species in this family, we measured nests from 49 species using museum collections. We observed that dome‐nesting species are present in dryer and hotter environments, in line with previous findings suggesting that domed nests are a specialisation for arid conditions. We also found evidence that nests with thicker walls are present in locations with lower precipitation and that solar radiation can influence the shape of domed nests; birds tend to build shorter and narrower domes in areas with high levels of solar radiation. Open nest architecture is also potentially influenced by wind speed, with longer and deeper nests in areas characterised by strong winds. Our results support the hypothesis that different climatic variables can drive the evolution of specific aspects of nest architecture and contribute to the diversity of nest shapes we currently observe. However, climatic variables account only for a small fraction of the observed structural variation, leaving a significant portion still unexplained.

## Graphical abstract

Bird nests play a crucial role in the survival and fitness of eggs, nestlings, and incubating parents, but the role of climate variables on the evolution and diversity of avian nest traits is still largely unknown. Previous studies have focused on (i) single species and few environmental variables or (ii) multiple species and general nest morphology. In our study, we explore the relationship between several climatic variables and specific nest traits in a family of birds, Thraupidae (commonly known as tanagers). Thraupidae represents an ideal clade for our study as species belonging to this family vary considerably in their nest architecture. We found evidence that nests with thicker walls are present in locations with lower precipitation and that solar radiation can influence the shape of domed nests; birds tend to build shorter and narrower nests in areas with high levels of solar radiation. Open nest architecture is also potentially influenced by wind speed, with longer and deeper nests in areas characterized by strong winds.

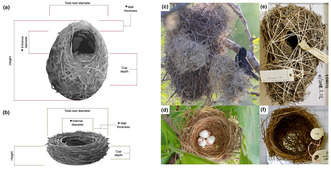

## 1 INTRODUCTION

Nest architecture among birds is diverse, and the climatic conditions in habitats where nests are built are likely to have played an important role in driving the evolutionary diversity of the nest characteristics we currently observe (Perez et al., 2020). Bird nests provide protection for eggs and nestlings not only from predators and parasites but also from harsh climatic conditions such as extreme temperatures or strong winds (Deeming, 2023; Perez et al., 2020; Scott‐Baumann & Morgan, 2015). Sub‐optimal climatic conditions inside nests can lead to abnormal development or even death of the embryos (Deeming et al., 2020; Mainwaring & Hartley, 2013; Ritz et al., 2005). Indeed, bird embryos can only partially control their temperatures and have a very limited temperature range (36–40°C) for ideal development (Du & Shine, 2015; Martin, 2008). Despite the importance of nests in reproduction, the study of drivers of variation in specific nest traits at a broad scale has been quite limited. Furthermore, there has been little previous investigation of the extent to which specific nest characteristics are influenced by particular environmental variables. Identifying the environmental drivers of nest architecture is crucial if we aim to understand how nest diversity evolved and how birds vary their construction behaviours to match their environment.

To maintain ideal temperatures inside nests, birds may perform specific behaviours such as fanning their wings to cool the eggs or incubating for longer to regulate the egg temperature (Durant et al., 2019; Mainwaring & Hartley, 2013). However, these behaviours are very energetically demanding, especially while incubating because parents already have less available foraging time (Deeming, 2011; Walsberg & Voss‐Roberts, 2015). Most small‐sized passerines spend between 20% and 40% of the daytime away from their nests; large non‐passerines species are more consistent in incubating for long periods of time during the day, but take longer breaks equalising the proportion of time spent away from the nest as in small passerine birds (Skutch, 1962). Given that birds spend a considerable amount of time away from their nests, these structures are likely to be crucial for protecting embryos from extreme climatic conditions. For instance, at least two laboratory experiments have shown that both dummy eggs and real eggs cool down more slowly in thicker nests (Akresh et al., 2017; Lambrechts & Caro, 2022). Likewise, it was observed that nest size and wall thickness influence nest thermal conductance (well‐insulated nests have low conductance; Heenan, 2013). Furthermore, the choice of nesting material also plays a role in nest conductance, particularly when exposed to precipitation, as certain types of material can accelerate the drying process (Heenan et al., 2015).

In passerines, three general types of nests can be found: (i) open nests which are simple, cup‐shaped structures, (ii) domed nests have a spherical shape, with a roof and a small entrance and (iii) cavity nests are cup or domed nests placed inside tree holes or rocky walls, or in the ground (Collias & Collias, 1984). Among passerines, open‐nesting species are common, and they occur more often in northern temperate regions, while dome‐nesting species are more common in the Tropics (Collias, 1997; Collias & Collias, 1984). Studies have suggested that domed nests could provide protection from extreme climatic environments and could be a type of specialisation in arid regions (Duursma et al., 2018; Martin et al., 2017; McEntee et al., 2018; Medina, 2019). In the case of the bay‐capped wren spinetail (*Spartonoica maluroides*), a species that builds both open and domed nests, nest type was linked to vegetation density, and domed nests were built in areas characterised by less dense and more sparse vegetation than open nests (Cardoni et al., 2017). Hence, there is potentially a role for aridity and temperature in the evolution of domed nests.

Specific nest traits have also been found to be linked to environmental variables. In certain species, birds have been observed adjusting the lining, diameter and height of their nests based on the prevailing climate or the specific stage of the nesting season in which they are constructing it (e.g. Janiga & Višňovská, 2004; Mainwaring & Hartley, 2008; Walsh et al., 2010). It has also been observed that captive zebra finches (*Taeniopygia guttata*), a dome‐nesting species, adjust the amount of lining and change the size of their nests in response to experimental ambient temperature changes (Campbell et al., 2018). Another study on captive zebra finches showed that their nests are built with a higher number of cotton strings at cooler ambient temperatures than at warmer temperatures to increase temperature inside the nests; however, at their second breeding attempt, birds used the same amount of strings of their previous successful nest independently of the ambient temperature (Edwards et al., 2020). In other species, such as the black‐throated blue warblers (*Setophaga caerulescens*), females at higher elevations (which typically have lower temperatures) build nests with thicker walls than females at lower elevations (Smith et al., 2018). The rufous hummingbird (*Selasphorus rufus*) also builds its nest at lower heights in Spring than in Summer when the levels of solar radiation near the ground are higher (Horvath, 1964). Additionally, seasonal changes in nest traits were observed in the nests of blue tits (*Cyanistes caeruleus*), where, as spring advances and the temperature increases, females decrease the amount of nesting material in the inner cups (Mainwaring et al., 2012; Mainwaring & Hartley, 2008). Lastly, the Hawaiian honeycreeper (*Hemignathus virens virens*) changes the density of the nest's walls depending on the habitat in which it builds its nests; in the warm and wet rainforest, nest walls are porous – allowing the nest to dry quickly – while in the cold upland savannah, the nest walls are dense, protecting the eggs from the outside environment (Kern & Van Riper, 1984). Nest characteristics are still understudied in many species even though there has been some increase in attention to this area in recent years; species such as the blue tit and the common starling (*Sturnus vulgaris*), as well as other species that nest inside nest boxes, have been favoured for study due to logistical convenience (Clark & Mason, 1985; Mainwaring & Hartley, 2008; Mainwaring, Hartley, et al., 2014).

To date, most studies that have explored the link between climatic pressures and nest architecture have focused on general nest types rather than nest traits, few climatic variables (temperature and/or humidity) and/or have included a single or few species of birds (Malzer & Hansell, 2017; Perez et al., 2020). Research on climatic data and nest traits has focused mainly on European and Australian species, and studies on South American birds are considerably fewer despite being the area with the highest diversity of bird species (Cerezo & Deeming, 2016; Gaston & Blackburn, 1997; Heenan et al., 2015; Lees et al., 2020; see also Deeming, 2023, for a detailed review). The 19th‐ and early‐20th‐century expeditions in the southern hemisphere contributed to an increase in the number of Neotropical ornithological specimens (especially type specimens that are linked to the scientific name) in European and North American museums offering an incredible resource to explore the diversity of specific nest traits at a macroevolutionary scale, including more detailed aspects of nest morphology and species from a wide geographic range (Heenan et al., 2015; Joseph, 2011; Rinkert et al., 2021; Russell et al., 2013).

In this study, we explore whether specific climatic variables have driven the evolution of nest traits in a family of birds, the tanagers (Thraupidae). With 382 species inhabiting predominantly Neotropics, Thraupidae is the second‐largest family of birds (Burns et al., 2014; Winkler et al., 2020). This family is ideal for exploring how nest architecture evolves because it comprises species that present a wide variation in nest construction including species that build open, domed or even partially domed nests, or that use cavity nests facultatively (Vanadzina, Street, & Sheard, 2023; Winkler et al., 2020). We visited museum collections, measured nest traits of different open‐ and dome‐nesting species belonging to this family, and used a broad range of climatic data to understand how environmental factors shaped nest evolution in this clade at a macroevolutionary scale.

## 2 MATERIALS AND METHODS

### 2.1 Nest data collection

We visited two of the largest nest collections in Europe, the Museums für Naturkunde in Berlin (Germany) with 1500 nests, and the Natural History Museum in Tring (United Kingdom) with more than 4000 nests (‘Birds | Museum für Naturkunde’, n.d.; Malhotra, 2021). We measured 89 nests (32 from the Berlin collection and 57 from the Tring collection; 35 domed nests and 54 open nests) belonging to 49 species (38 open‐nesting species and 11 dome‐nesting species) of the 382 extant species in the Thraupidae family (representing 12.83% of extant species and 23.22% of genera; Winkler et al., 2020). All the nests were collected in South and Central America from 1861 to 1971.

**FIGURE 1:**
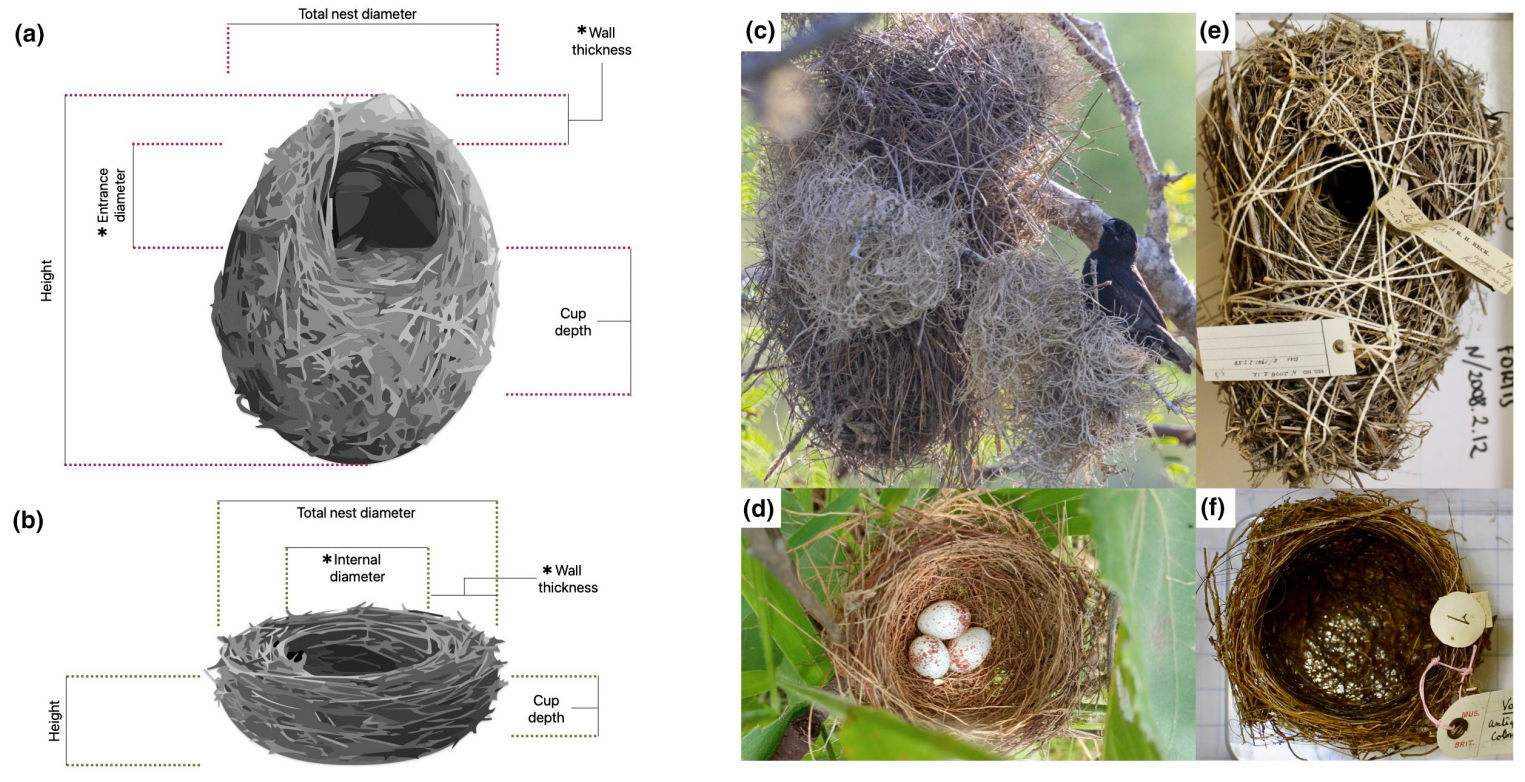
Schematic diagram of the structure and measurements of domed (a) and open (b) nests; asterisks indicate measurements that were taken at different points and then averaged (see Appendix S1 for more details, drawings by Daniela Perez). Photographs of medium ground finch (*Geospiza fortis*) nests in the field (c) and in the museum (e); and blue‐black grassquit (*Volatinia jacarina*) nests in the field (d) and in the museum (f). Photo credits: (c) Todd Dixon – ML569819351 and (d) Carlos Otávio Gussoni – ML307543541, both from Macaulay Library at the Cornell Lab of Ornithology; (e) and (f) S.C., courtesy of the Trustees of the Natural History Museum, London.

Nests included in our study were first selected based on their quality. We excluded any damaged nests and, when possible, we measured more than one nest per species (min 1, max 6 nests). We used digital callipers to measure nest height, total nest diameter, internal (entrance in domed nests) diameter, wall thickness and cup depth (Appendix S1 for further details on nest measurements; Figure 1). Given many measurements were highly correlated, we selected a subset of representative variables after checking for associations among variables (Figure S1).

### 2.2 Environmental variables

We collected nest location information from each nest record, but when precise coordinates were not available in the collection data, we used the general location given on the nest label to extract coordinates from a place close by that is part of the reported range of the species; if only a general area was provided, we selected a random point in the closest natural area in the species breeding range indicated on the Handbook of Birds of the World Alive (HBW, Winkler et al., 2020). In our analysis, we also considered the month in which nests were collected; however, for half of the nests (*N* = 45), the date of nest collection was not provided on the label, so we relied on the HBW (Winkler et al., 2020) and extracted information on the month during which most nests of that species were likely to be built. For this subset of nests, we also averaged climatic data over the entire breeding period and repeated the analyses that had significant results with the previous approach, to confirm our results. For each species, we also sourced the adult mass (average of male and female body mass) from the public dataset Avonet (Tobias et al., 2022), given that nest characteristics are likely to be linked to the size of the builder.

To test whether variation in nest traits is linked to environmental variables, we downloaded climatic data from the public dataset ERA5‐Land from the Copernicus Climate Change Service (Muñoz Sabater, 2021), for the location where the nest was collected. We extracted the following climatic variables: temperature at 2 m, total precipitation, surface net solar radiation and 10 m u‐component (eastward wind) and v‐component (northward wind; Muñoz Sabater, 2021; Table S1).

For each nest, we downloaded 10 years of hourly climatic data (from 1960 to 1969) for the month in which the nest was collected; as nest collection was scattered among a time range of more than 100 years, a 10‐year sample of climatic data was chosen as this time span has been shown to be sufficient for accounting the long‐term effects of climatic variables on avian patterns (e.g. Arct et al., 2022; Li et al., 2021; Saraux et al., 2011). We then extracted the maximum, minimum and mean values per year and averaged them across all years. To reduce the number of variables and redundancy for temperature, precipitation and solar radiation, we performed three principal component analyses (PCAs; Table S2). The first principal components obtained from the PCAs of temperature, precipitation and solar radiation variables are referred to as temperature (PC), precipitation (PC) and radiation (PC) respectively.

### 2.3 Statistical analyses

All analyses were conducted using RStudio (Version 2022.12.0, available from RStudio Team, 2015). To test the relationship between nest measurements and climatic data, considering the phylogenetic relatedness among species, we ran Bayesian Regression Models using the R package *brms* separately for open and domed nests (Bürkner, 2021). We divided our analysis into species that build open and those that build domed nests (Figure S2) because the architecture of these two nest types is too different to compare measurements in the same analyses, but we used similar model structures for both categories. We conducted a further combined analysis for wall thickness measurements, including both open and domed nest datasets, as this particular trait exhibits the highest level of comparability between the two nest types.

We ran four models for the open nest category and five models for the dome‐nesting category. Nest measurements were the response variables, and temperature (PC), precipitation (PC), radiation (PC) and wind speed were the predictors in the models as well as the log‐transformed mass of species. Species mass was log‐transformed to correct for a strong positive skew. All predictors included in the models were tested to ensure that they were not highly correlated with each other (see Appendix S1 for further details).

**FIGURE 2:**
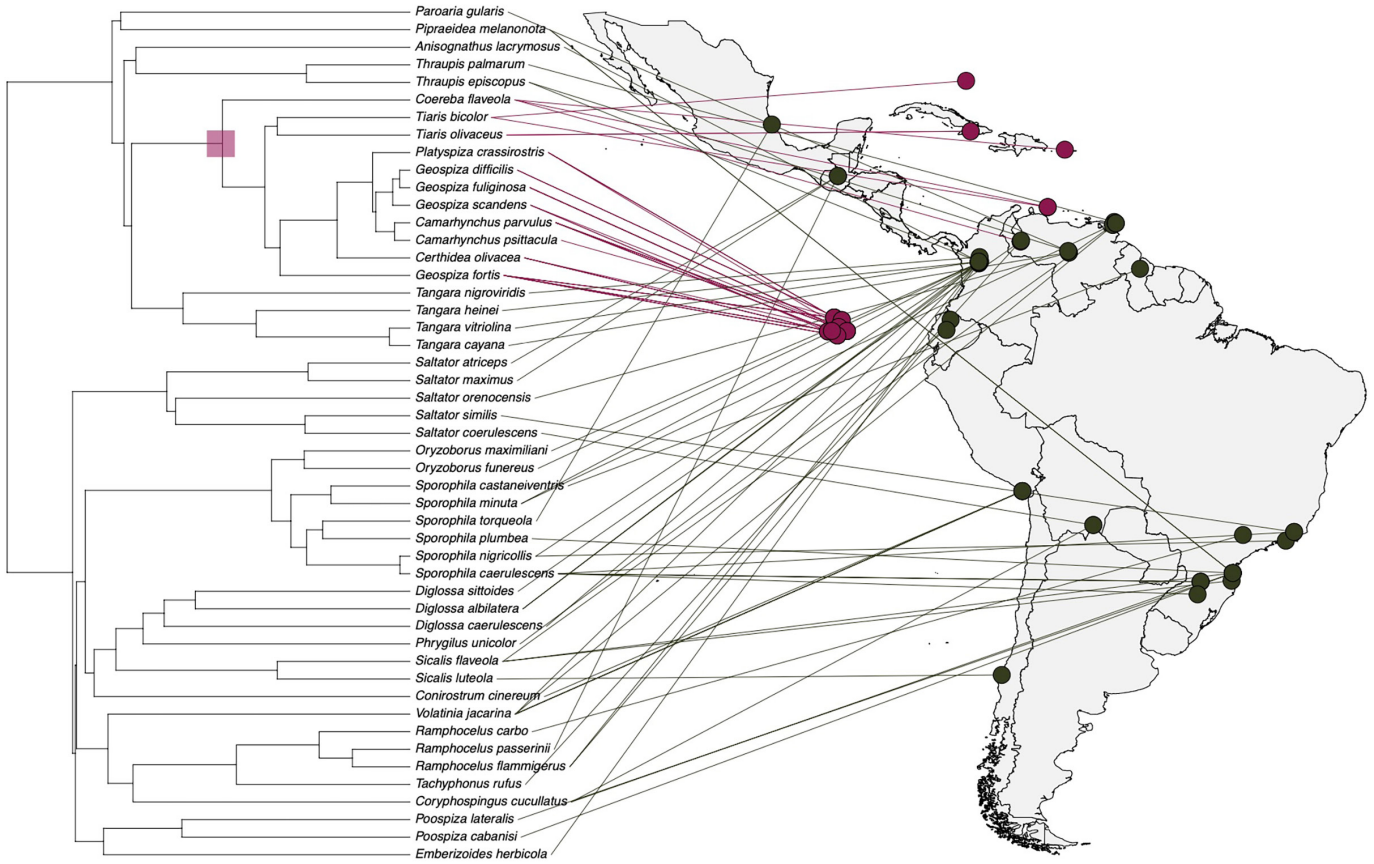
Maximum clade credibility (MCC) phylogenetic tree of the nests included in the study; the purple square highlights the single origin of the dome‐nesting clade. The map shows location of collection of the nests included in this study; domed nests are represented in purple; open nests are represented in dark green (*n* = 49 species, *n* = 89 nests).

To account for the potential influence of phylogenetic relatedness between species on nest traits, we generated a maximum clade credibility tree (MCC, Figure 2) from birdtree.org using 1000 phylogenies and the ‘Phangorn’ package (Schliep, 2011). The species identity and phylogenetic effect were included as random factors, given we had multiple nests for 40% of species. For the open nest data subset, four models with different nest measurements as response variables were executed: nest height, internal diameter, wall thickness and cup depth (Table S3). For the domed‐nest data subset, the same four models were run for the open nests and an additional model with total nest diameter as the response (Table S5). In the domed‐nest subset, nest height values were log‐transformed to facilitate model convergence. All model structures were checked for collinearity to keep all variance inflation factors below the recommended threshold of five (Lüdecke et al., 2019).

We performed BRMS models with a Gaussian distribution, running four chains in parallel with a warm‐up of 1000 iterations followed by 60,000 iterations; effective sample sizes are reported for each model in the Appendix S1. Predictors are considered to have an effect if confidence intervals do not overlap 0. For models that showed evidence of an effect using the MCC tree, Bayesian regression analyses were conducted on a set of 100 phylogenetic trees to test the effect of phylogenetic uncertainty. In addition to reporting the estimates for each model based on the MCC tree, we also generated 95% highest posterior density intervals (HPD) for the estimates across 100 trees using the ‘coda’ Package (Plummer et al., 2006), these trees used the Hackett backbone (Jetz et al., 2012). For each Bayesian regression analysis conducted on a set of 100 phylogenetic trees, we also reported ‘marginal’ and ‘conditional’ *R* ^2^ values, which correspond to the portion of overall variance accounted for by the fixed effects and the portion of overall variance explained by both the fixed and random effects respectively (Nakagawa & Schielzeth, 2013).

## 3 RESULTS

**FIGURE 3:**
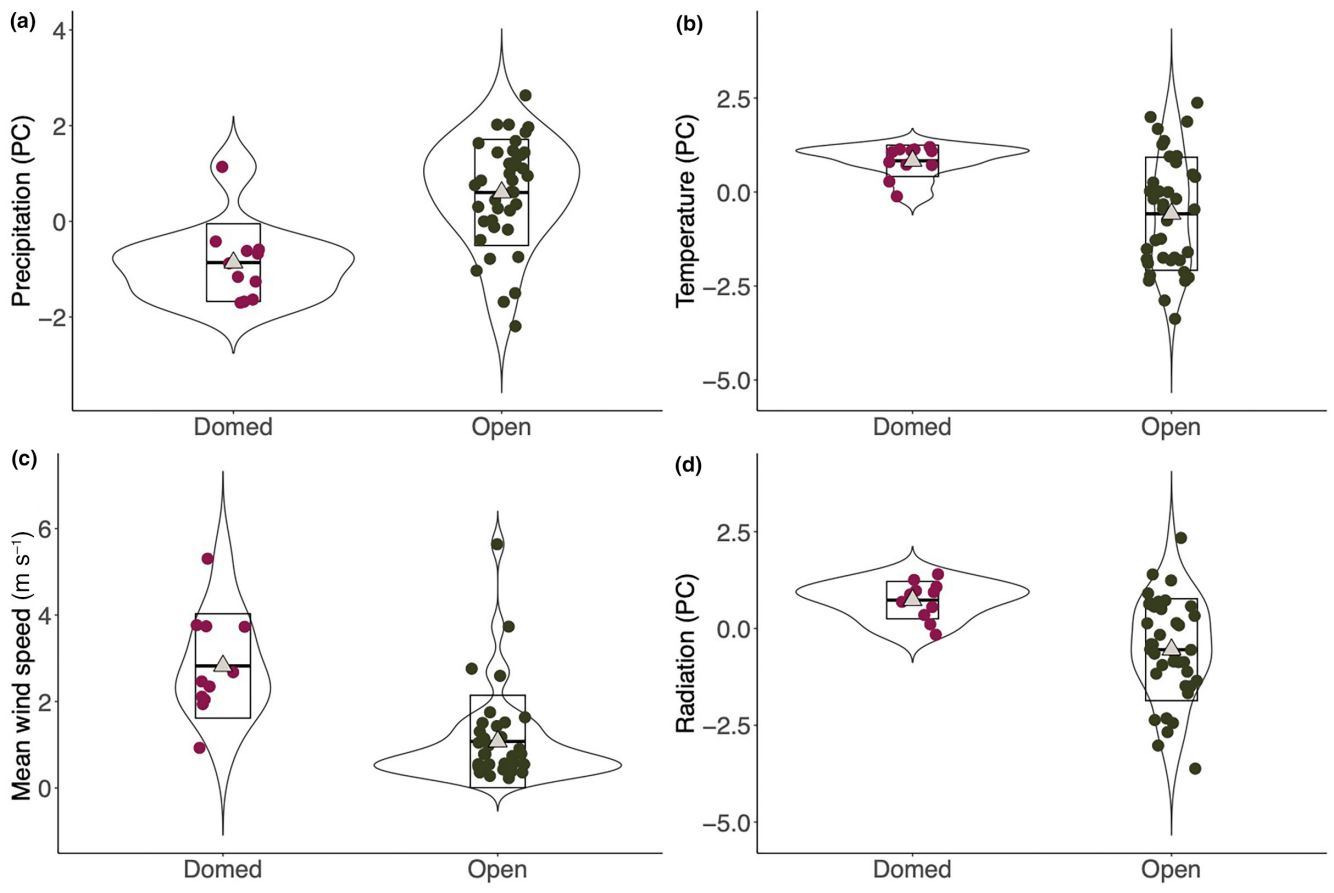
Differences in Precipitation (PC, a), Temperature (PC, b), mean wind speed (c), and Radiation (PC, d) between open‐ and dome‐nesting species (*n* = 11 dome‐nesting species, *n* = 38 open‐nesting species). The points represent each species, the triangles represent the mean and the line within each box represents the median. The upper and lower lines of the box indicate the minimum and maximum quantiles. High values of Radiation (PC) indicate high maximum and mean solar radiation levels; high values of Precipitation (PC) indicate high maximum and mean precipitation values; high values of Temperature (PC) indicate high maximum, mean and minimum temperatures.

Species that build open nests were found across broader ranges of temperature and solar radiation than domed nests (Figure 3). Dome‐nesting species were found in drier, more windy places with higher solar radiation. Given there is only one origin of dome‐nesting in the clade (Figure 2), this is just a descriptive pattern and we did not perform phylogenetically corrected statistical analyses on the climate of dome‐ and open‐nesting species due to lack of independent origins of dome‐nesting, which result in no statistical power.

**FIGURE 4:**
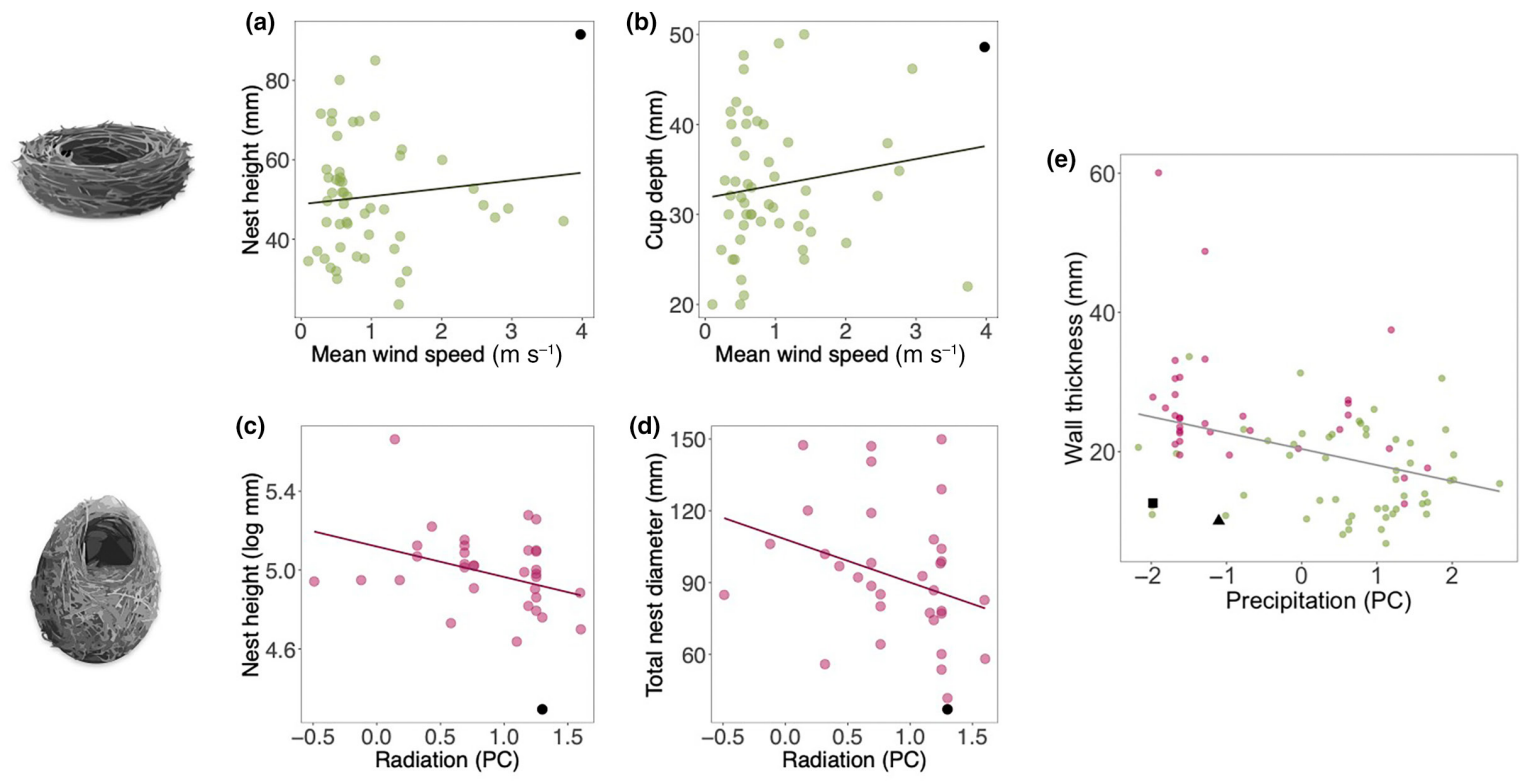
Relationship between nest measurements and environmental variables in open‐ (a, b), dome‐ (c, d) and combined open‐ and dome‐ (e) nesting species (*n* = 34 domed nests, *n* = 53 open nests). The data points represent nests, the lines represent the predictions from the model; the black point in open nest plots highlights the nest belonging to *Sicalis flaveola*; the black point in domed nest plots highlights the nest belonging to the species *Certidea olivacea*; in the open and domed combined plot, the black square highlights the nest belonging to the species *C. olivacea* and the triangle highlights the nest belonging to *S. flaveola*. Both are considered influential points and analyses without these data are presented in the Appendix S1. High values of Radiation (PC) indicate high maximum and mean solar radiation levels; high values of Precipitation (PC) indicate high maximum and mean precipitation values. Drawings by Daniela Perez.

For open nests, we found evidence that species in locations with stronger winds have longer nests (estimate = 7.63, [95% CI 1.74–13.51], Figure 4a, Table S3) with deeper cups (estimate = 4.56, [95% CI 1.18–7.96], Figure 4b, Table S3). The consistency of the effect found was verified by conducting the same analysis across 100 trees from which we generated 95% HPD intervals (Table S3). However, the effect of wind on nest height and cup depth weakens when a nest belonging to the species *Sicalis flaveola* is removed (cup depth: estimate = 3.43, [95% CI −0.35 to 7.20]; nest height: estimate = 4.30, [95% CI −2.11 to 10.74], Table S4). This nest is not identified as an outlier, but it is a highly influential point. We found that, when this point is removed, a negative association between precipitation and wall thickness is detected (estimate = −1.25, [95% CI −2.46 to −0.03, Table S4]), and although there is a tendency, such effect is absent when using the whole dataset (estimate = −1.34, [95% CI −2.77 to 0.10], Table S3).

For domed nests, we found that species in environments with high solar radiation build shorter (estimate = −0.22, [95% CI −0.41 to −0.03], Figure 4c, Table S5) and narrower (estimate = −23.60, [95% CI −43.84 to −3.17], Figure 4d, Table S5) nests than species that live in locations with low solar radiation. However, the detected effect of solar radiation on nest height decreases when a nest belonging to the species *Certidea olivacea* is excluded (estimate = −0.15, [95% CI −0.34 to 0.04], Table S6); this data point is not an outlier but is a highly influential point. Consequently, the effects we have detected are likely to be weak. The effect of solar radiation on total nest diameter is still present when the nest belonging to the species *C. olivacea* is excluded (estimate = −21.93, [95% CI −43.85 to −0.29], Table S6). A negative effect of precipitation on wall thickness was also found; species that live in areas with low precipitation build nests with thicker walls (Estimate = −2.20, [95% CI −4.20 to −0.05], Table S5). The effect of precipitation on wall thickness is also present when the nest belonging to the species *C. olivacea* is excluded (Estimate = −2.15, [95% CI −3.84 to −0.33], Table S6). For these results, we executed the same analysis across 100 trees and generated the 95% HPD intervals for the estimates across 100 trees.

As we found an influence of precipitation on wall thickness in both open and domed nests, and this measure is comparable across nest types, we combined both datasets and re‐run the analysis. The result is in line with the results from the two separate datasets, with nest walls decreasing in thickness with increasing precipitation (estimate = −1.55, [95% CI −2.76 to −0.34], Figure 4e, Table S7). This effect is present also when points identified previously as influential are removed (estimate = −1.77, [95% CI −2.77 to −0.77], Table S8). Interestingly, when these nests are excluded, an effect of wind speed on wall thickness is also found, with thicker walls in nests collected in locations with stronger winds (estimate = 1.19, [95% CI 0.26–2.13], Figure S3, Table S8).

We found an effect of log‐transformed body mass on some nest traits (Figure S4). For open nests, nests are longer and have bigger internal diameters and thicker walls in species with higher average mass (Table S3). These effects are still present when the nest belonging to the species *S. flaveola* is removed (Table S4). For domed nests, nests are wider and have bigger entrance diameters in species with higher average mass (Table S5). These effects are present also when the nest belonging to the species *C. olivacea* is excluded (Table S6).

When combining open and domed nest subsets, nests have thicker walls in species with higher average mass (Table S7).

Given that nests were collected in different years, we also tested whether the year of collection influenced nest measurements, aiming to identify potential biases attributed to the age of the specimens. No discernible differences were found in nest measures across years (Figure S5). Lastly, the influence of the climatic variables on the nest parameters observed in our initial analysis persisted, albeit to a lesser extent, when using an average of the climatic data of the entire breeding period instead of information on the month during which most nests of that species were likely to be built (Figure S6).

## 4 DISCUSSION

Our results suggest that climatic variables appear to influence the evolution of some nest traits in both open and domed nests. We found that all climatic variables except for temperature were linked to one or several nest traits. In open nests, longer nests are found in places with stronger winds; in domed nests, smaller size is linked to higher solar radiation. In both open and domed nests, we found evidence that higher precipitation is related to narrower wall thickness. We also found that the size of the bird has strong effects on some nest traits in both open‐ and dome‐nesting species, supporting the idea that nests are important not only for protecting the eggs and nestlings from external climatic factors but also for giving structural support to the incubating parents.

Despite having a tropical distribution, our results show that Thraupidae species can be found in a wide variety of environments, ranging from very cold places at high altitudes such as the Andean highlands (average minimum temperature 9.6°C) to very dry and hot islands in South and Central America (average minimum temperature 22.7°C). Our data shows that domed nests in this clade are distributed in locations with relatively higher solar radiation, higher temperature and more wind (Figure S7). This is similar to the result of an analysis of Australian passerine species, which showed that dome‐nesting species are more abundant in hotter and drier environments (Duursma et al., 2018). Given that there was only a single origin of dome‐nesting species in our dataset, we cannot test whether domed nests have independently evolved in arid environments, but the patterns reported support the idea that domed nests could have evolved as a specialisation to such conditions (Duursma et al., 2018). Interestingly, nearly all domed nests in our dataset except one were collected on islands (possibly because these specimens were collected during scientific expeditions focused on island biogeography) while only 3 open nests of 54 were collected on islands. However, in contrast to the high number of domed nests collected on islands in our dataset, a recent study by Vanadzina, Street, and Sheard (2023), which included more than 4000 species of passerines, showed that birds inhabiting islands are significantly less likely to build domed nests. It is possible that the prevalence of dome‐nesting species on islands depends on the family studied and the type of island considered, in our case islands were always tropical.

We found that open‐nesting species that build their nests in locations with strong winds have longer nests and deeper cups than species that build their nests in areas with lighter winds. This pattern is relatively weak (especially after removing the influential nest), suggesting that there are other variables besides climate‐driving nest size evolution. A similar pattern, however, was observed in an intra‐specific study, supporting our findings: in yellow warblers (*Dendroica petechia*), a species that includes individuals that breed in cold locations and others that breed in warmer locations, larger and longer open nests are found in areas experiencing higher wind speeds (Rohwer & Law, 2010). Wind is known to have an adverse effect on reproduction and the damaging effects of wind on nests have been observed in several within‐species studies (Facemire et al., 1990; Fisher et al., 2011; Kim & Monaghan, 2005; Pinowski et al., 2006; Sidis et al., 1994). In the presence of wind, nests experience elevated heat loss rates that can impact incubating parents, eggs and nestlings (Heenan & Seymour, 2012). An experimental study in which nests were positioned inside a wind tunnel showed that nest mass has the biggest role in cooling rates inside the nests, lessening the impact of air movement (Gray & Deeming, 2017). As most of the studies on wind speed and nest traits have focused on different nest characteristics from those in this study – such as nest orientation or placement (Ferguson & Siegfried, 1989; Hadley, 1969; Long et al., 2009) – our result contributes to this poorly explored field of study. It has been shown previously that nest size increases in colder climates (Perez et al., 2020; Vanadzina, Street, Healy, et al., 2023), and, although temperature was not linked to nest size in our dataset, we now show that wind speed could also be an important driver of nest size.

Nest depth could also serve as a safeguard against the risk of eggs and nestlings being dislodged during gusts of strong wind. Several species are known to adopt strategies to prevent eggs from falling from the nest during strong wind gusts, such as changing the orientation of the nest entrance in domed nests or building the nests on more stable and thicker branches (Collias & Collias, 1984; Schaefer, 1977). Furthermore, Zheng et al. (2022) showed experimentally that the Chinese penduline tit (*Remiz consobrinus*) buries its eggs in domed nests to prevent them from rolling out during strong gusts of wind. Both thermoregulatory and mechanical benefits are not exclusive, and it is possible that longer nests with a deeper cup serve multiple functions in locations with intense winds.

When the datasets comprising both open and domed nests are merged, and influential nests are excluded, an influence of wind speed on wall thickness was observed. Nests with thicker walls tend to be found in areas with strong winds, thus reinforcing the hypothesis that nest traits play a crucial role in protecting eggs and nestlings from the adverse (mechanical and thermoregulatory) effects of strong wind gusts. The influence of wall thickness on nest thermal characteristics, however, is still under debate; Gray and Deeming (2017) showed that the mass of the nest (and the composition of nesting materials) rather than the wall thickness impacts nest insulation buffering the effects of air movement. Further investigations will help in understanding the connections among nest mass, wall thickness and the insulatory properties of the nests.

In domed nests, we found a link between nest height and total nest diameter and solar radiation, specifically finding that nests were shorter and narrower in environments with elevated solar radiation levels. This relationship is consistent with the hypothesis that domed nests are specialised to help protect against high levels of solar intensity (Collias & Collias, 1984). Shorter nests also imply shallower nests, serving as an indicator of the overall nest size. In our sample, nest height varies from 7 cm (in *Certhidea olivacea*) to almost 29 cm (in *Geospiza scadens*). The link between solar radiation and nest height of domed nests can be attributed to the fact that shorter nests expose less surface to direct sunlight and thus reduce heat absorption, preventing overheating inside the nest. According to Collias and Collias (1984), birds can prevent excessive heat build‐up within their nests by either diminishing the nest's size and insulation, or in some cases, by eliminating the nest altogether.

The study of the role of solar radiation on specific nest traits in different nest types is still under investigation as past research focused particularly on nest site selection and entrance orientation, especially in open‐nesting species; for example, previous studies have highlighted the significant impact of sunlight on the well‐being and survival of parents, eggs and nestlings (Tomiałojć & Neubauer, 2017; Walsberg, 1981). Solar radiation has been found to influence nest orientation in dot‐fronted woodpeckers (*Veniliornis frontalis*) and rufous hornero (*Furnarius rufus*) with birds adjusting the entrance orientation in colder environments at the beginning of the breeding season in order to get more solar radiation, thereby increasing the nest temperatures and decreasing the humidity (Schaaf, 2020a, 2020b). Moreover, it has been observed that cavity nests located in the shade have higher nestling survival rates (Charter et al., 2010). The role of solar radiation on nest survival is so crucial that, in some species, birds abandon their nests when solar intensity reaches a critical level (Amat & Masero, 2009). Our results highlight the impact of solar radiation on avian nests, suggesting that solar radiation has possibly driven the evolution of nest height and size in dome‐nesting species.

We also found a relationship between precipitation and wall thickness, with domed nests showing thicker walls in areas characterised by lower precipitation rates. This effect is present also when open and domed nests are combined (Figure 4e). The connection between nest wall thickness and precipitation remains uncertain, as past research has yielded conflicting results. For example, in the blackbird (*Turdus merula*), a species that builds open nests, Mainwaring, Deeming, et al. (2014) found no relationship between wall thickness and precipitation; however, in a comprehensive study that included more than 200 open‐nesting species, it was observed that, in areas with high precipitation rates, birds build nest walls using less‐insulating materials, possibly in order to speed up the drying process (Heenan et al., 2015). Similarly, common amakihi (*Hemignathus virens virens*), an open‐nesting species that occurs both in the rainforest and the savannah, builds more porous nests in the rainforest to accelerate the drying process after heavy rain; moreover, nests built by this species have denser but not thicker walls at higher elevation (Kern & Van Riper, 1984). Our results suggest that thinner walls in areas with high precipitation could provide better insulation as they dry more quickly, retain less water and help maintain a more suitable environment for eggs, nestlings and incubating parents. In our study, we did not investigate the composition of the materials used in the construction of nests, as the nests were museum specimens. Prior studies have shown the significance of the nest composition in relation to water absorbance, highlighting how different materials can affect absorbency and the duration required for the nest to dry (Biddle et al., 2019).

Lastly, we found that bird mass is a strong predictor of some nest traits in both open and domed nests, but it depends on the variable tested. In open nests, nest height, internal diameter and wall thickness are positively correlated with the mass of the bird species; in domed nests, total nest diameter and entrance diameter show a positive correlation with bird mass. Our findings validate previous research highlighting the influence of bird mass on nest traits in both open and domed nests. It has been shown recently that larger species build deeper cups and larger nests (Vanadzina, Street, Healy, et al., 2023). A study on Australian passerines also showed that larger bird species build nests of bigger size compared to smaller species and that nest thickness is related to the mass of the parent as it potentially provides structural support during incubation (Heenan & Seymour, 2011). A comprehensive comparative analysis of nest traits and female body mass showed that internal diameter in open nests is highly correlated with body mass, suggesting that birds construct cups that fit their bodies closely, which could minimise heat loss (Deeming, 2013).

We acknowledge some limitations of our study, such as the fact that nests were collected and conserved in museums for many years before being measured, as this certainly could have changed the original shape of the nests and potentially could have obscured or weakened patterns detected. However, no discernible connection was found between the year of collection and the measurements (Figure S5). Furthermore, nests were collected in various years and locations, often lacking precision, which complicates the acquisition of accurate climate data corresponding to the specific localities. It has been shown that nest composition is also influenced by location, as it depends on the available resources in the environment (Briggs & Deeming, 2016); because of the value of the museum specimens, an analysis of the material composition of nests was not feasible in this study.

Future research could conduct experimental or field tests to explore the mechanisms behind the patterns we have identified, such as why there are deeper cups in windy environments and shorter nests in areas with high radiation. This approach would enable us to quantify the magnitude of the benefits that these potential adaptations may provide. We believe our findings are also relevant in the context of climate change, as they show an influence of climatic variables on nest traits in both open and domed nest types. Some bird species may possess nest structures that are well suited for specific environments. However, as temperatures change dramatically, these structures may no longer be optimally adapted to their surroundings, negatively influencing the species' survival. Further research is needed to determine if this is the case.

To conclude, our results show evidence that at least four different nest traits – wall thickness, nest height, total nest diameter and cup depth – might have evolved in response to variations in precipitation, wind speed, and solar radiation. Although some associations are weak, they support the idea that the whole nest structure responds to multiple selective pressures from different climatic variables, and variation in different traits could be product of specific adaptations.

## AUTHOR CONTRIBUTIONS

**Silvia Colombo:** Data curation (equal); formal analysis (equal); investigation (equal); methodology (equal); software (equal); validation (equal); visualization (equal); writing – original draft (lead); writing – review and editing (equal). **Kevin D. Newman:** Formal analysis (supporting); software (supporting); writing – review and editing (supporting). **Naomi E. Langmore:** Conceptualization (supporting); supervision (supporting); validation (supporting); writing – review and editing (supporting). **Claire J. Taylor:** Conceptualization (supporting); formal analysis (equal); investigation (equal); methodology (equal); software (equal); supervision (equal); validation (equal); visualization (equal); writing – original draft (equal); writing – review and editing (equal). **Iliana Medina:** Conceptualization (lead); formal analysis (equal); investigation (equal); methodology (equal); project administration (equal); software (equal); supervision (lead); validation (equal); visualization (equal); writing – original draft (equal); writing – review and editing (equal).

## FUNDING INFORMATION

This research was supported by APSF Australasian Pacific and Science Foundation grant to IM and Melbourne Research Scholarship, University of Melbourne, to SC. Open access publishing facilitated by The University of Melbourne, as part of the Wiley – The University of Melbourne agreement via the Council of Australian University Librarians.

## CONFLICT OF INTEREST STATEMENT

The authors declare no conflicts of interest.

## Supporting information


Appendix S1

## Data Availability

Data and code used to generate our results have been archived in figshare (https://doi.org/10.6084/m9.figshare.24589587.v3).
