# Supplementary material for: The role of climatic variables on nest evolution in tanagers
Source: Ecol Evol. 2024 Apr 1;14(4):e11168. doi: 10.1002/ece3.11168 (PMC10985373; doi:10.1002/ece3.11168)
Supplement: Supplementary file 1 — Appendix S1 [file ECE3-14-e11168-s001.pdf]

SUPPLEMENTARY MATERIALS

Nest measurements

Nest height: In open nests, the measurement of length extended from the entrance (the rim of the cup) to the bottom of the nest, while in domed nests, height was determined from the top of the nest to the bottom.

Cup depth: For both nest types, the depth of the cup was measured from the entrance to the bottom, employing a slender ruler.

Total nest diameter: In open nests, width was defined as the broadest part of the nest, while in domed nests, width was gauged along the base of the entrance hole.

Internal (entrance for domed nests) diameter: In open nests, internal diameter was ascertained by measuring the internal diameter both vertically and horizontally, followed by an averaging of these two measurements. In domed nests, the entrance diameter was ascertained by measuring the entrance diameter both vertically and horizontally, followed by an averaging of these two measurements.

Wall thickness: Determined by measuring the nest's thickness at four distinct points (evenly spaced at 90 degrees from each other around the entrance hole) and then averaging these four measurements.

All measurements besides cup depth were taken using digital calipers and all nests were photographed in a standardized manner.

Collinearity

A subset of the nest measurements was selected after checking for correlation between variables (Figure S1). A threshold of 0.7 was chosen to discard one of the highly correlated variables, resulting in the nest width variable in the open nest subset, being discarded.

We used the R package "Performance" and the command *check_model* to check for collinearity between predictor variables. The degree of collinearity is determined using the variance inflation factor (VIF), where a VIF value of less than 5 is deemed acceptable for individual predictors (Lüdecke et al., 2019). The number of response variables was chosen on the basis of VIF value, when a high correlation between two variables was highlighted one of the variables was discarded. In both open and domed nest subsets, enough variation in the predictors in all models was detected to avoid multicollinearity issues.
One nest in the domed nest category and one in the open nest category were discarded as they were considered outliers.

Table S1. Climatic variables included in this study (Muñoz Sabater, 2021).

Climate variable	Description	Units
**2-meter temperature**	Temperature of air at 2 meters above the ground	Kelvin
**Total precipitation**	Water (including rain and snow) that falls to the ground	m (depth in meters of water equivalent)
**Surface net short-wave (solar) radiation**	Shortwave solar radiation that is reduced by the amount reflected by the ground	J m^{-2}
**10-meter U wind component (10u)**	Eastward component of the wind at a height of ten meters above the ground. Given the u and v components, the magnitude of the wind vector can be determined using the Pythagorean Theorem: wind speed= sqrt ($10u^2 + 10v^2$).	m s^{-1}
**10-meter V wind component (10v)**	Northward component of the wind at a height of ten meters above the ground	m s^{-1}

Table S2. Loadings and proportion of variance obtained from the Principal Component Analysis on temperature, precipitation, and solar radiation variables.

Weather variable	PC1	PC2
Max Temperature	-0.486	-0.822
Min Temperature	-0.583	0.557
Mean Temperature	-0.651	0.114
**Proportion of variance explained**	**77.66**	**22.18**
Max Precipitation	-0.701	0.111
Min Precipitation	0.107	0.993
Mean Precipitation	-0.705	0.040
**Proportion of variance explained**	**59.43**	**33.10**
Max Solar Radiation	-0.700	0.058
Min Solar Radiation	0.229	0.962
Mean Solar Radiation	-0.677	0.265
**Proportion of variance explained**	**59.48**	**32.44**

Table S3. BRMS results for open nests. Predictors are Temperature (PC), Precipitation (PC), Radiation (PC), mean wind speed (mean wind), and the log-transformed mass (log(mass)) of the species.

**Response**	**Predictor**	**Estimate**	**Est. Error**	**l-95% CI_{MCC}**	**u-95% CI_{MCC}**	**Rhat**	**l-95% HPD $interval_{100}$**	**u-95% HPD $interval_{100}$**	**Bulk ESS**	**Tail ESS**	**Marg. R2**	**Cond. R2**
**Cup depth**	Intercept	15.75	7.81	-0.04	30.88	1.00			205551	165553	0.229	0.298
	Temperature (PC)	-1.17	0.80	-2.75	0.41	1.00			250754	194422		
	**Mean wind**	**4.56**	**1.72**	**1.18**	**7.96**	**1.00**	**4.548**	**4.603**	**207734**	**189040**		
	log(mass)	3.67	2.28	-0.78	8.26	1.00			249603	177482		
	Precipitation (PC)	2.16	1.16	-0.14	4.45	1.00			238444	187723		
	Radiation (PC)	-0.71	0.85	-2.38	0.96	1.00			275118	187438		
**Nest height**	Intercept	5.04	14.95	-24.20	35.15	1.00			141812	144815	0.301	0.459
	Temperature (PC)	-1.31	1.43	-4.12	1.51	1.00			172717	177871		
	**Mean wind**	**7.63**	**2.99**	**1.74**	**13.51**	**1.00**	**7.563**	**7.716**	**154707**	**171960**		
	**log(mass)**	**12.50**	**4.40**	**3.62**	**21.05**	**1.00**	**12.205**	**2.688**	**166204**	**151199**		
	Precipitation (PC)	2.02	2.02	-1.94	5.99	1.00			187426	180451		
	Radiation (PC)	-1.36	1.56	-4.39	1.76	1.00			168659	169038		
**Internal diameter**	Intercept	16.11	6.87	2.41	29.53	1.00			190632	166457	0.594	0.654
	Temperature (PC)	-0.67	0.69	-2.03	0.70	1.00			208350	186879		
	Mean wind	1.94	1.45	-0.90	4.80	1.00			190091	185563		
	**log(mass)**	**12.81**	**2.01**	**8.86**	**16.82**	**1.00**	**12.732**	**12.863**	**226053**	**173001**		
	Precipitation (PC)	0.84	0.98	-1.08	2.77	1.00			223816	186158		
	Radiation (PC)	0.04	0.73	-1.48	1.39	1.00			272224	188431		
**Wall thickness**	Intercept	6.58	7.25	-7.00	21.50	1.00			82815	131142	0.277	0.688
	Temperature (PC)	0.67	0.57	-0.44	1.80	1.00			90064	147138		
	Mean wind	-0.90	1.21	-3.25	1.51	1.00			79318	137801		
	**log(mass)**	**4.48**	**2.10**	**0.14**	**8.42**	**1.00**	**4.177**	**4.820**	**97088**	**139850**		
	Precipitation (PC)	-1.34	0.73	-2.77	0.10	1.00			181890	172868		
	Radiation (PC)	-0.38	0.58	-1.52	0.76	1.00			202296	184941		

Table S4. BRMS results for open nests excluding the nest belonging to the species *Sicalis flaveola*. Predictors are Temperature (PC), Precipitation (PC), Radiation (PC), mean wind speed (mean wind), and the log-transformed mass (log(mass)) of the species.

Response	Predictor	Estimate	Est. Error	l-95% CI_{MCC}	u-95% CI_{MCC}	Rhat	l-95% HPD $interval_{100}$	u-95% HPD $interval_{100}$	Bulk ESS	Tail ESS	Marg. R2	Cond. R2
**Cup depth**	Intercept	18.24	8.06	2.11	33.96	1.00			173394	158026	0.192	0.278
	Temperature (PC)	-1.15	0.79	-2.71	0.41	1.00			232289	186300		
	Mean wind	3.43	1.92	-0.35	7.20	1.00			184161	180942		
	log(mass)	3.19	2.32	-1.35	7.84	1.00			212144	170434		
	Precipitation (PC)	2.03	1.16	-0.25	4.31	1.00			216289	187634		
	Radiation (PC)	-0.55	0.85	-2.23	1.12	1.00			248862	186766		
**Nest height**	Intercept	12.81	14.91	-16.04	43.04	1.00			144901	143295	0.257	0.426
	Temperature (PC)	-1.21	1.38	-3.91	1.51	1.00			185014	181169		
	Mean wind	4.30	3.26	-2.11	10.74	1.00			167618	179053		
	**log(mass)**	**10.98**	**4.32**	**2.17**	**19.28**	**1.00**	**10.621**	**11.205**	**169550**	**155799**		
	Precipitation (PC)	1.67	1.95	-2.17	5.54	1.00			193788	182333		
	Radiation (PC)	-0.99	1.50	-3.91	1.98	1.00			181731	174959		
**Internal diameter**	Intercept	15.40	7.16	0.99	29.26	1.00			181066	166028	0.595	0.654
	Temperature (PC)	-0.88	0.69	-2.24	0.49	1.00			233915	193890		
	Mean wind	2.24	1.67	-1.04	5.54	1.00			204229	186996		
	**log(mass)**	**12.95**	**2.06**	**8.94**	**17.08**	**1.00**	**12.861**	**13.006**	**210976**	**174862**		
	Precipitation (PC)	0.87	0.99	-1.09	2.83	1.00			241479	188765		
	Radiation (PC)	-0.08	0.74	-1.54	1.38	1.00			250597	186790		
**Wall thickness**	Intercept	3.66	6.77	-9.36	17.34	1.00			101616	140681	0.310	0.814
	Temperature (PC)	0.83	0.49	-0.14	1.77	1.00			109375	152933		
	Mean wind	0.75	1.11	-1.42	2.97	1.00			103117	150107		
	**log(mass)**	**4.96**	**1.97**	**0.98**	**8.73**	**1.00**	**4.422**	**5.302**	**116726**	**149410**		
	**Precipitation (PC)**	**-1.25**	**0.61**	**-2.46**	**-0.03**	**1.00**	**-1.271**	**-1.103**	**169583**	**169285**		
	Radiation (PC)	-0.44	0.52	-1.47	0.58	1.00			132302	169904		

Table S5. BRMS results for domed nests. Predictors are Temperature (PC), Precipitation (PC), Radiation (PC), mean wind speed (mean wind), and the log-transformed mass (log(mass)) of the species.

Response	Predictor	Estimate	Est. Error	l-95% CI$_{MCC}$	u-95% CI$_{MCC}$	Rhat	l-95% HPD interval$_{100}$	u-95% HPD interval$_{100}$	Bulk ESS	Tail ESS	Marg. R2	Cond. R2
**Cup depth**	Intercept	107.74	28.41	51.84	164.57	1.00			77682	77297	0.390	0.436
	Temperature (PC)	3.83	6.41	-8.60	16.74	1.00			78121	78721		
	log(mass)	-17.08	9.61	-36.48	1.70	1.00			78868	77054		
	Precipitation (PC)	2.43	3.09	-3.67	8.48	1.00			79117	76899		
	Radiation (PC)	-10.64	7.35	-25.17	3.88	1.00			78740	77264		
	Mean wind	-3.05	2.22	-7.46	1.33	1.00			79590	78115		
**log(nest height)**	Intercept	4.45	0.39	3.66	5.19	1.00			78919	78230	0.440	0.471
	Temperature (PC)	0.13	0.08	-0.04	0.29	1.00			78569	78115		
	log(mass)	0.19	0.13	-0.06	0.46	1.00			78923	77012		
	Precipitation (PC)	-0.05	0.04	-0.13	0.03	1.00			78877	77398		
	**Radiation (PC)**	**-0.22**	**0.09**	**-0.41**	**-0.03**	**1.00**	**-0.225**	**-0.221**	**77782**	**77456**		
	Mean wind	-0.00	0.13	-0.06	0.46	1.00			77254	77890		
**Total nest diameter**	Intercept	-4.15	46.30	-97.20	86.66	1.00			74784	76898	0.335	0.788
	Temperature (PC)	14.32	8.86	-3.28	31.76	1.00			74104	76002		
	**log(mass)**	**33.63**	**16.21**	**1.98**	**66.22**	**1.00**	**32.054**	**33.668**	**74734**	**77421**		
	Precipitation (PC)	-2.46	4.16	-10.83	5.60	1.00			74915	76285		
	**Radiation (PC)**	**-23.60**	**10.27**	**-43.84**	**-3.17**	**1.00**	**-23.833**	**-23.189**	**76009**	**76882**		
	Mean wind	2.22	2.86	-3.44	7.81	1.00			75016	75855		
**Entrance diameter**	Intercept	-10.60	28.88	-69.44	45.52	1.00			77912	76673	0.417	0.590
	Temperature (PC)	-3.72	5.50	-14.61	7.07	1.00			73910	77341		
	**log(mass)**	**24.18**	**10.20**	**4.58**	**45.33**	**1.00**	**23.873**	**24.782**	**77262**	**76910**		
	Precipitation (PC)	-0.55	2.45	-5.48	4.16	1.00			72461	76603		
	Radiation (PC)	-8.08	6.37	-20.69	4.42	1.00			76919	78089		
	Mean wind	1.10	1.75	-2.44	4.48	1.00			75504	77606		
**Wall thickness**	Intercept	-2.85	19.11	-40.84	35.15	1.00			77846	77287	0.336	0.787
	Temperature (PC)	3.56	2.80	-2.13	8.86	1.00			74923	71498		
	log(mass)	9.60	6.79	-3.96	23.07	1.00			77874	77831		
	**Precipitation (PC)**	**-2.20**	**1.05**	**-4.20**	**-0.05**	**1.00**	**-2.216**	**-2.186**	**78857**	**75968**		
	Radiation (PC)	-6.51	3.27	-12.79	0.09	1.00			75630	76194		
	Mean wind	0.28	0.75	-1.16	1.81	1.00			78654	75980		

Table S6. BRMS results for domed nests excluding the nest belonging to the species *Certidea olivacea*. Predictors are Temperature (PC), Precipitation (PC), Radiation (PC), mean wind speed (mean wind), and the log-transformed mass (log(mass)) of the species.

Response	Predictor	Estimate	Est. Error	l-95% CI_{MCC}	u-95% CI_{MCC}	Rhat	l-95% HPD $interval_{100}$	u-95% HPD $interval_{100}$	Bulk ESS	Tail ESS	Marg. R2	Cond. R2
**Cup depth**	Intercept	109.38	28.42	53.05	165.86	1.00			78539	78456	0.381	0.431
	Temperature (PC)	1.14	7.29	-13.02	15.69	1.00			79460	78794		
	log(mass)	-17.50	9.56	-36.73	1.27	1.00			77931	77922		
	Precipitation (PC)	2.69	3.16	-3.56	8.89	1.00			77569	77639		
	Radiation (PC)	-8.55	7.61	-23.56	6.57	1.00			79616	77721		
	Mean wind	-2.64	2.29	-7.18	1.88	1.00			78457	77487		
**log(nest height)**	Intercept	4.52	0.38	3.72	5.24	1.00			77462	76963	0.388	0.421
	Temperature (PC)	0.03	0.09	-0.15	0.20	1.00			77452	76463		
	log(mass)	0.18	0.13	-0.07	0.45	1.00			76644	76047		
	Precipitation (PC)	-0.05	0.04	-0.12	0.03	1.00			78673	78241		
	Radiation (PC)	-0.15	0.10	-0.34	0.04	1.00			77766	78176		
	Mean wind	0.02	0.03	-0.04	0.07	1.00			76565	77590		
**Total nest diameter**	Intercept	-1.80	46.50	-96.09	89.22	1.00			76764	77672	0.392	0.621
	Temperature (PC)	11.95	10.18	-8.20	32.02	1.00			77808	78964		
	**log(mass)**	**32.96**	**16.16**	**1.27**	**65.61**	**1.00**	**31.326**	**33.080**	**75654**	**76710**		
	Precipitation (PC)	-2.40	4.26	-10.96	5.81	1.00			73882	75966		
	**Radiation (PC)**	**-21.93**	**10.98**	**-43.85**	**-0.29**	**1.00**	**-22.123**	**-21.419**	**77208**	**77450**		
	Mean wind	2.55	2.98	-3.39	8.43	1.00			76640	77791		
**Entrance diameter**	Intercept	-11.74	28.99	-71.10	44.55	1.00			77887	76664	0.423	0.585
	Temperature (PC)	-1.85	6.32	-14.31	10.71	1.00			75383	77262		
	**log(mass)**	**24.33**	**10.18**	**4.87**	**45.45**	**1.00**	**23.873**	**24.782**	**77488**	**76015**		
	Precipitation (PC)	-0.77	2.56	-5.93	4.14	1.00			74214	76964		
	Radiation (PC)	-8.96	6.61	-22.06	4.06	1.00			78671	78408		
	Mean wind	0.78	1.87	-2.98	4.38	1.00			76643	76124		
**Wall thickness**	Intercept	-0.32	18.31	-36.84	35.97	1.00			77007	77015	0.313	0.846
	Temperature (PC)	1.00	2.64	-4.45	5.98	1.00			73441	74644		
	log(mass)	8.81	6.50	-4.09	21.77	1.00			76845	76334		
	**Precipitation (PC)**	**-2.15**	**0.89**	**-3.84**	**-0.33**	**1.00**	**-2.163**	**-2.141**	**77165**	**77427**		
	Radiation (PC)	-4.75	2.96	-10.37	1.30	1.00			73827	76058		
	Mean wind	0.68	0.65	-0.58	2.02	1.00			77551	76291		

Table S7. BRMS results for open and domed nests combined. Predictors are Temperature (PC), Precipitation (PC), Radiation (PC), mean wind speed (mean wind), and the log-transformed mass (log(mass)) of the species.

Response	Predictor	Estimate	Est. Error	l-95% CI_{MCC}	u-95% CI_{MCC}	Rhat	l-95% HPD $interval_{100}$	u-95% HPD $interval_{100}$	Bulk ESS	Tail ESS	Marg. R2	Cond. R2
**Wall thickness**	Intercept	0.63	5.87	-10.81	12.30	1.00			142316	155073	0.274	0.748
	Temperature (PC)	0.56	0.55	-0.52	1.64	1.00			179835	179064		
	**log(mass)**	**6.25**	**1.87**	**2.54**	**9.89**	**1.00**	**6.066**	**6.332**	**146825**	**155642**		
	**Precipitation (PC)**	**-1.55**	**0.62**	**-2.76**	**-0.34**	**1.00**	**-1.580**	**-1.506**	**166499**	**171595**		
	Radiation (PC)	-0.55	0.68	-1.88	0.78	1.00			146201	173105		
	Mean wind	0.68	0.57	-0.43	1.81	1.00			175887	172084		

Table S8. BRMS results for open and domed nests combined excluding the nest belonging to the species *Certidea olivacea* and the nest belonging to the species *Sicalis flaveola*. Predictors are Temperature (PC), Precipitation (PC), Radiation (PC), mean wind speed (mean wind), and the log-transformed mass (log(mass)) of the species.

Response	Predictor	Estimate	Est. Error	l-95% CI_{MCC}	u-95% CI_{MCC}	Rhat	l-95% HPD $interval_{100}$	u-95% HPD $interval_{100}$	Bulk ESS	Tail ESS	Marg. R2	Cond. R2
**Wall thickness**	Intercept	0.21	5.52	-10.59	11.18	1.00			113683	151851	0.355	0.846
	Temperature (PC)	0.66	0.48	-0.28	1.60	1.00			158589	178678		
	**log(mass)**	**6.30**	**1.77**	**2.77**	**9.77**	**1.00**	**6.183**	**6.396**	**112888**	**152397**		
	**Precipitation (PC)**	**-1.77**	**0.51**	**-2.77**	**-0.77**	**1.00**	**-1.791**	**-1.746**	**157630**	**175442**		
	Radiation (PC)	-0.39	0.59	-1.56	0.77	1.00			131450	167884		
	**Mean wind**	**1.19**	**0.48**	**0.26**	**2.13**	**1.00**	**1.183**	**1.204**	**182337**	**172988**		

Supplementary figures

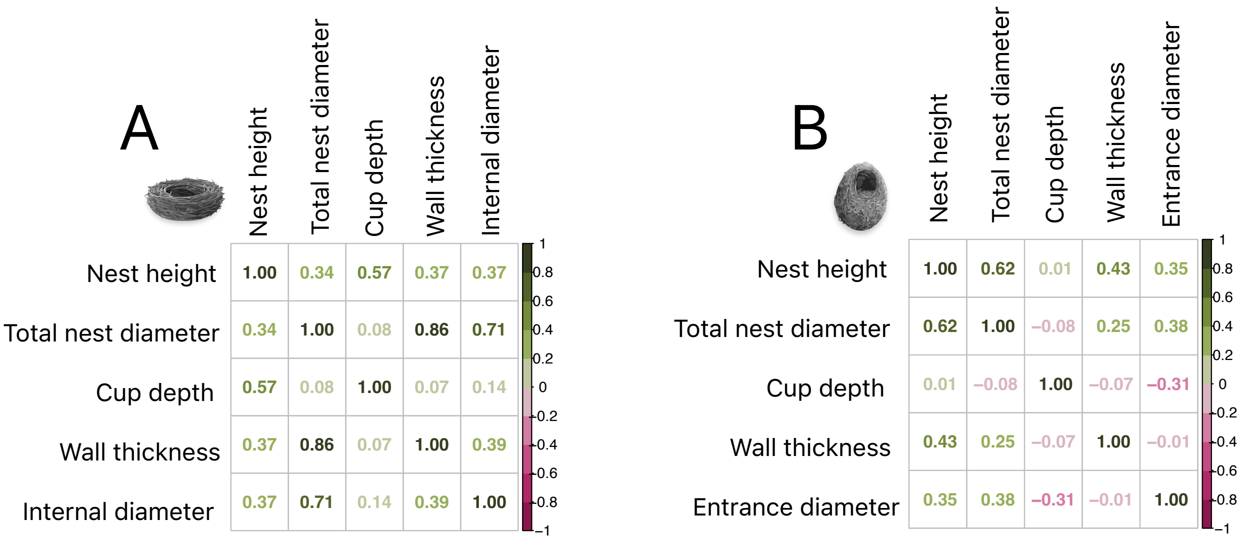

Figure S1. Correlation plots for open (A) and domed (B) nest measurements. Drawings by Daniela Perez.

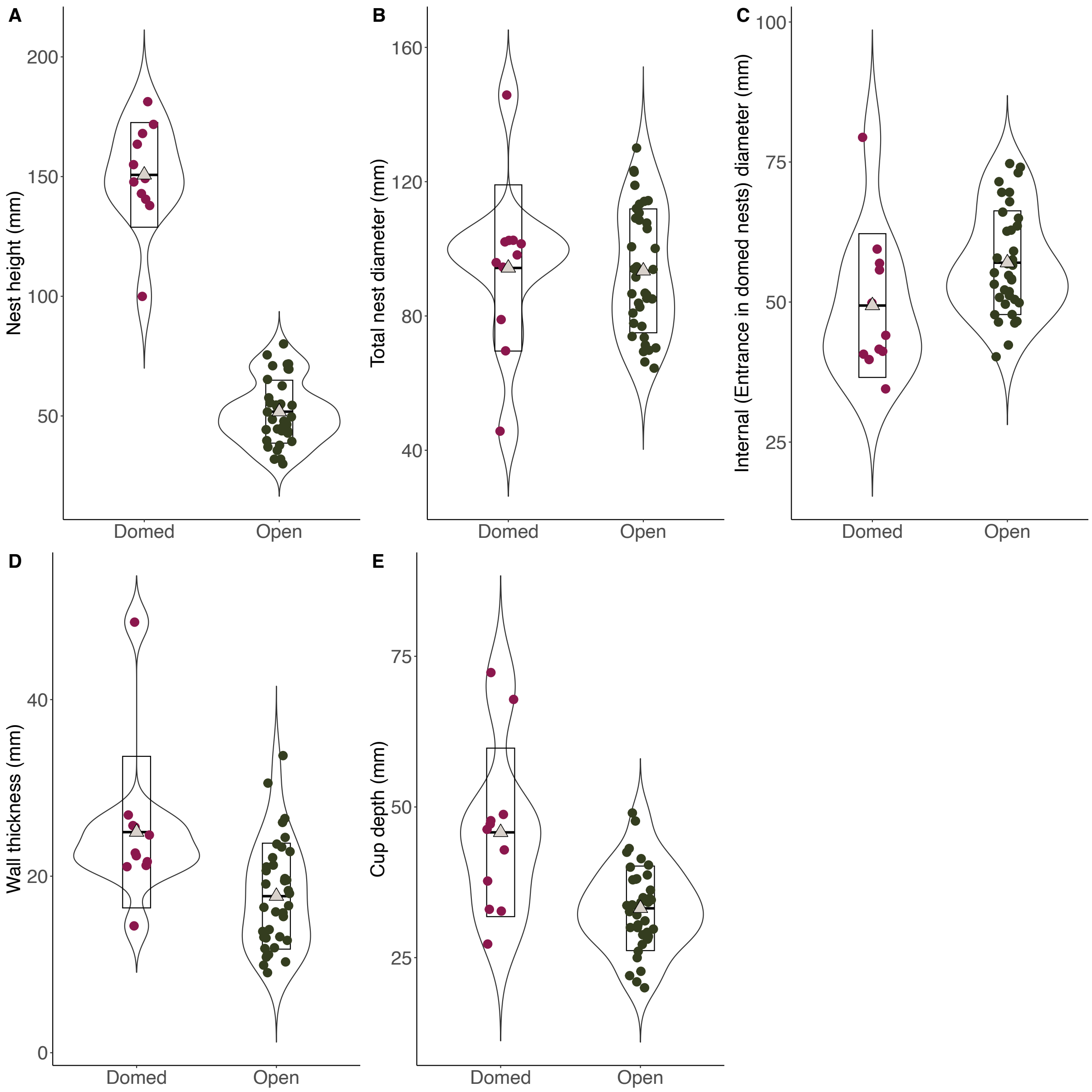

Figure S2. Nest trait measurements comparison between open and dome- nesting species (n = 11 dome-nesting species, n = 38 open nesting species). The points represent the raw data for each nest, the triangles represent the mean, and the line within each box represents the median. The upper and lower lines of the box indicate the minimum and maximum quantiles.

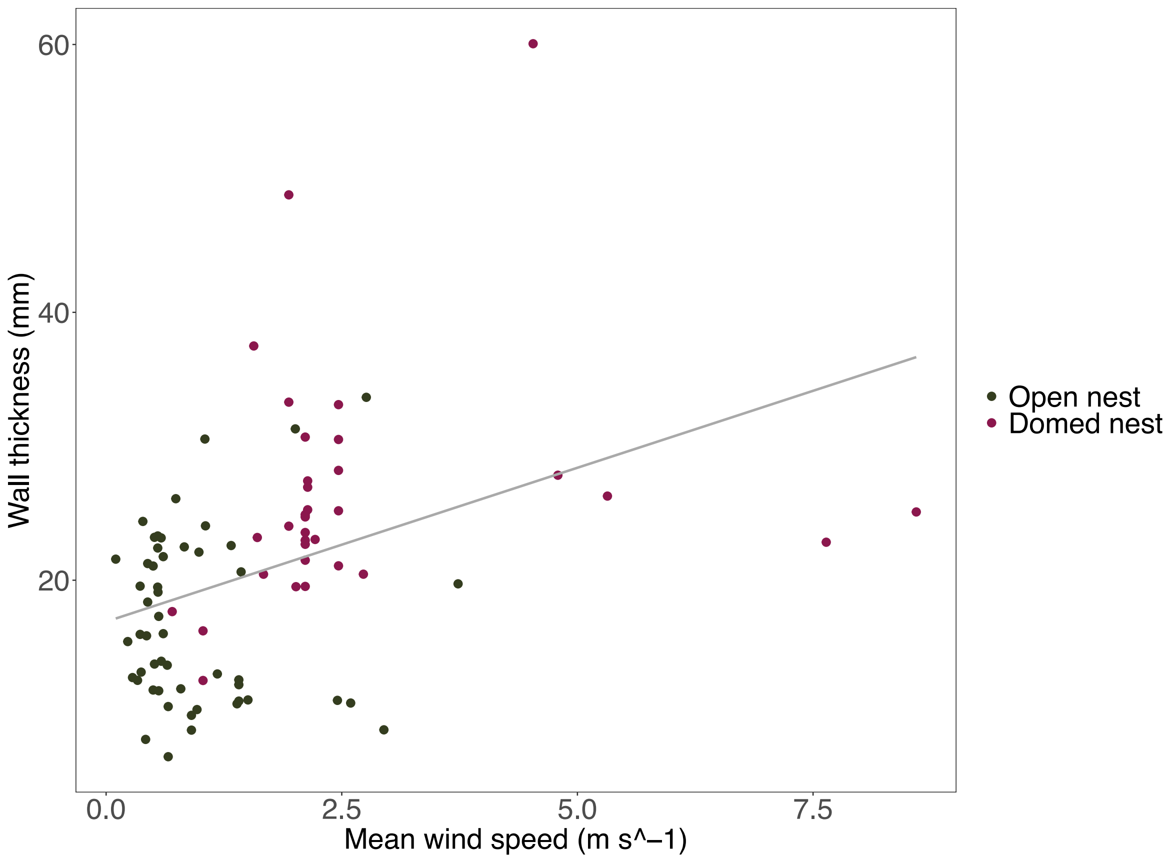

Figure S3. Relationship between wind speed and wall thickness (mm) combing data from open and domed nest subsets excluding the nests belonging to the species *Certidea olivacea* and the species *Sicalis flaveola* (n = 85 nests, n = 33 domed nests, n = 52 open nests).

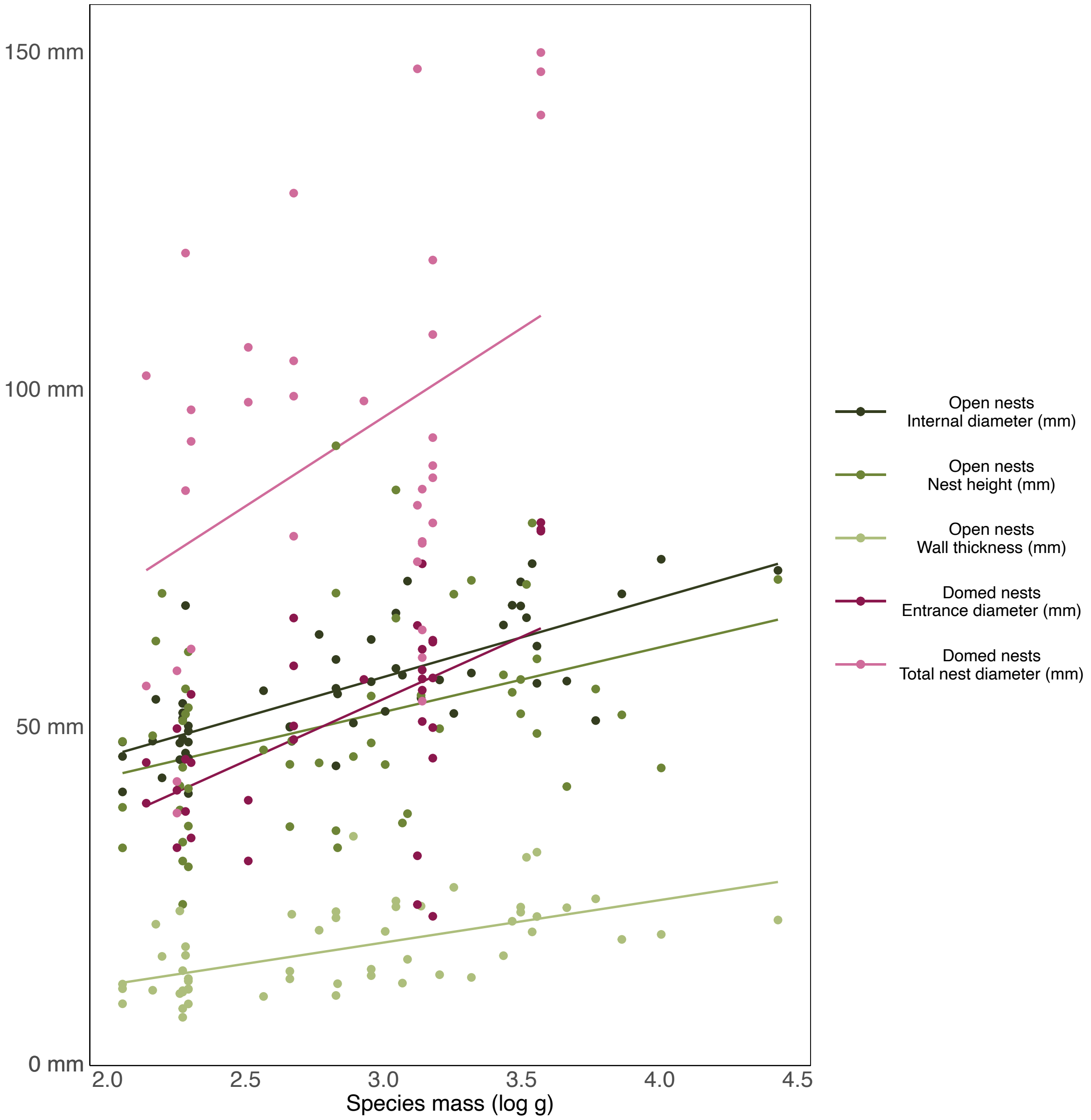

Figure S4. Correlation between nest traits and bird species mass (n = 89 nests, n = 35 domed nests, n = 54 open nests). Nest traits are expressed in millimeters, bird mass is log-transformed (log g). Nest traits associated with open nests are visualized in various shades of green, while those related to domed nests are represented in shades of purple. Each data point represents an individual nest measurement. Mass data (average of male and female body mass) was collected from the public dataset Avonet (Tobias et al., 2022).

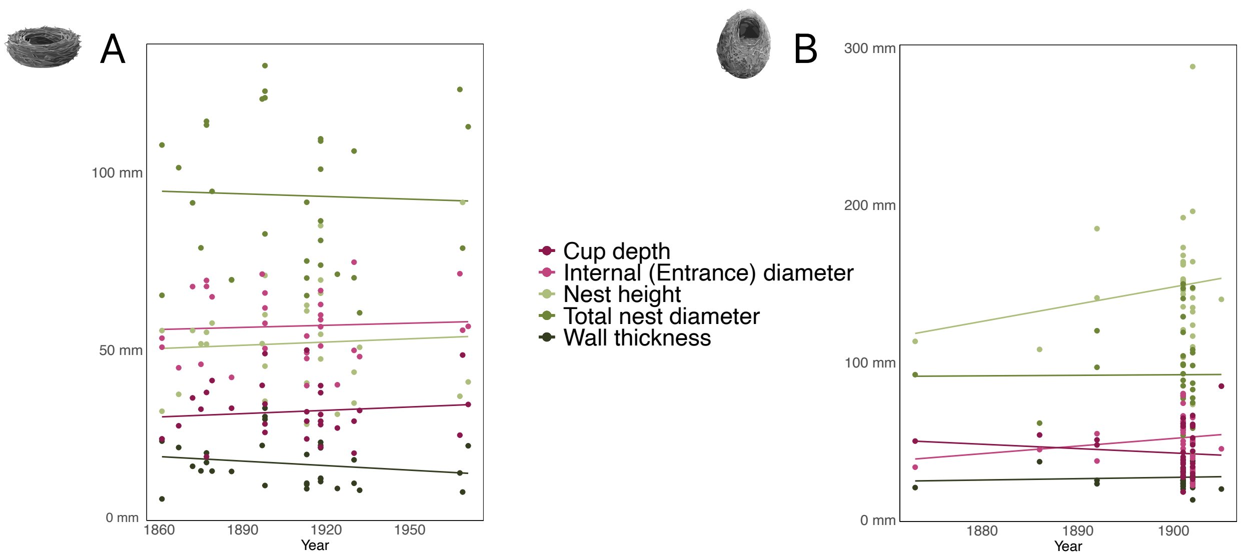

Figure S5. Relationship between year of collection and recorded measurements (n = 89 nests, n = 35 domed nests, n = 54 open nests). The year of collection for open nests spans from 1861 to 1971 (A); the year of collection for domed nests spans from 1873 to 1905 (B). Drawings by Daniela Perez.

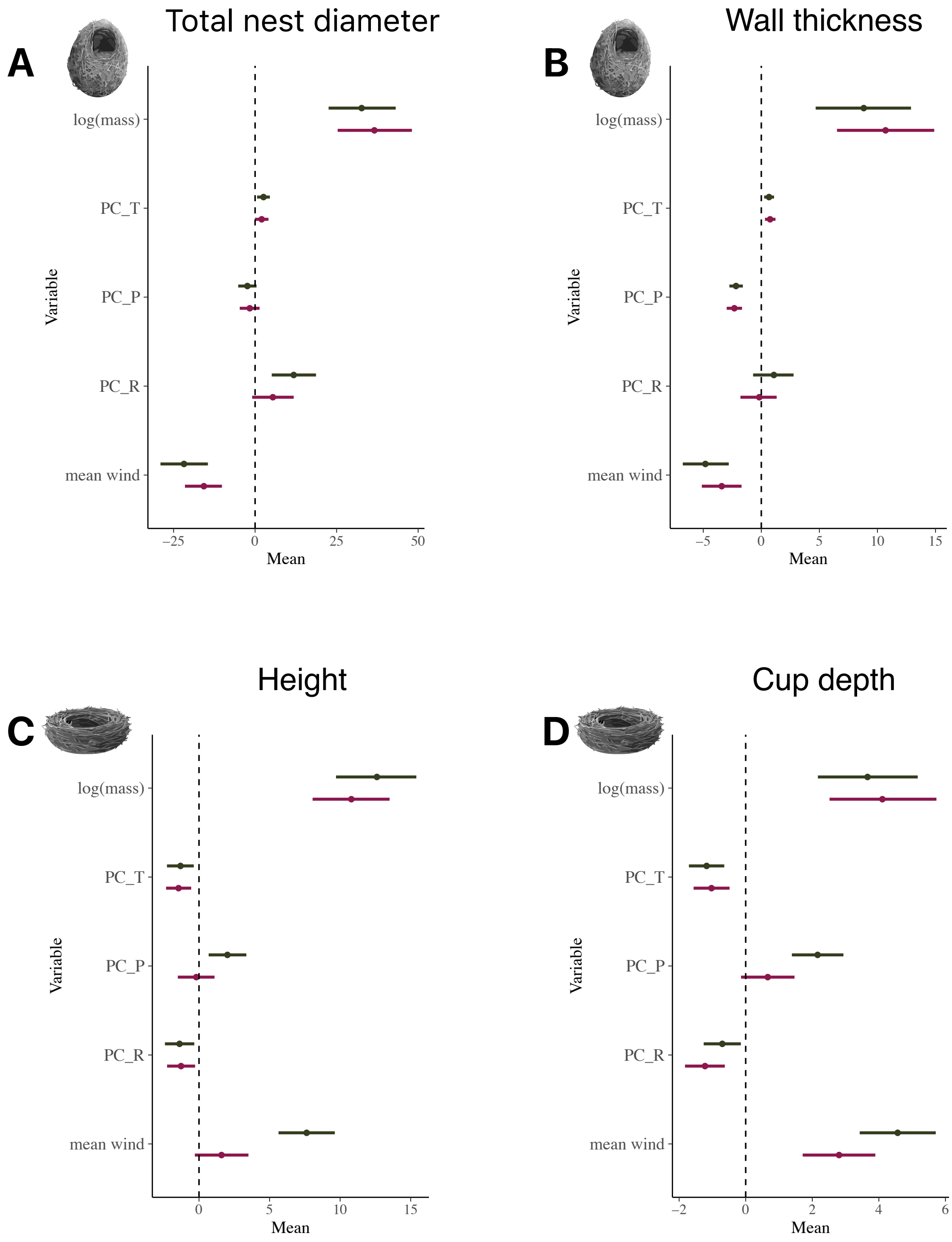

Figure S6. Posterior samples from the brms models for domed nests (A – B) and open nests (C – D) using climatic data using an average of the months of the breeding period for those nests whose month of collection was not provided (in purple) and using climatic data for the month during which most nests of that species were likely to be built (in green). Predictors are Temperature (PC), Precipitation (PC), Radiation (PC), mean wind speed, and the log-

transformed mass (log(mass)) of the species. Posterior samples for domed nests (A – B) exclude the nest belonging to the species *Certidea olivacea*.

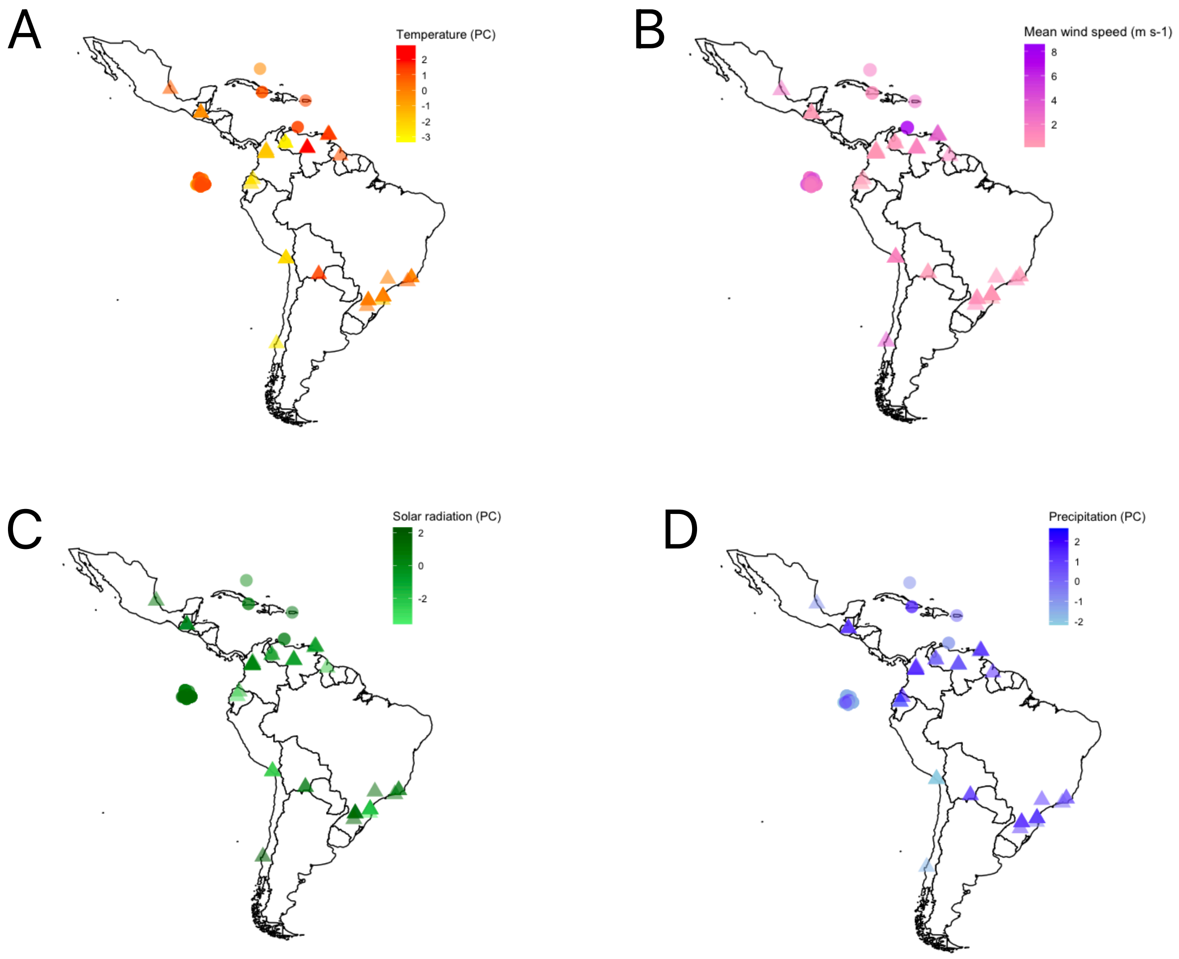

Figure S7. Nest distribution and climatic variables: Temperature (PC) (A); mean wind speed (B); Solar radiation (PC) (C); Precipitation (PC) (D). Domed nests are depicted with circles, open nests with triangles (n = 89 nests, n = 35 domed nests, n = 54 open nests).

REFERENCES

Lüdecke, D., Waggoner, P.D., Makowski, D., 2019. insight: A unified interface to access information from model objects in R. J Open Source Softw 4, 1412. https://doi.org/10.21105/joss.01412

Muñoz Sabater, J., 2021. ERA5-Land hourly data from 1950 to 1980. Copernicus Climate Change Service (C3S) Climate Data Store (CDS) https://doi.org/10.24381/cds.e2161bac

Tobias, J.A., Sheard, C., Pigot, A.L., Devenish, A.J.M., Yang, J., Sayol, F., Neate-Clegg, M.H.C., Alioravainen, N., Weeks, T.L., Barber, R.A., Walkden, P.A., MacGregor, H.E.A., Jones, S.E.I., Vincent, C., Phillips, A.G., Marples, N.M., Montaño-Centellas, F.A., Leandro-Silva, V., Claramunt, S., Darski, B., Freeman, B.G., Bregman, T.P., Cooney, C.R., Hughes, E.C., Capp, E.J.R., Varley, Z.K., Friedman, N.R., Korntheuer, H., Corrales-Vargas, A., Trisos, C.H., Weeks, B.C., Hanz, D.M., Töpfer, T., Bravo, G.A., Remeš, V., Nowak, L., Carneiro, L.S., Moncada R., A.J., Matysioková, B., Baldassarre, D.T., Martínez-Salinas, A., Wolfe, J.D., Chapman, P.M., Daly, B.G., Sorensen, M.C., Neu, A., Ford, M.A., Mayhew, R.J., Fabio Silveira, L., Kelly, D.J., Annorbah, N.N.D., Pollock, H.S., Grabowska-Zhang, A.M., McEntee, J.P., Carlos T. Gonzalez, J., Meneses, C.G., Muñoz, M.C., Powell, L.L., Jamie, G.A., Matthews, T.J., Johnson, O., Brito, G.R.R., Zyskowski, K., Crates, R., Harvey, M.G., Jurado Zevallos, M., Hosner, P.A., Bradfer-Lawrence, T., Maley, J.M., Stiles, F.G., Lima, H.S., Provost, K.L., Chibesa, M., Mashao, M., Howard, J.T., Mlamba, E., Chua, M.A.H., Li, B., Gómez, M.I., García, N.C., Päckert, M., Fuchs, J., Ali, J.R., Derryberry, E.P., Carlson, M.L., Urriza, R.C., Brzeski, K.E., Prawiradilaga, D.M., Rayner, M.J., Miller, E.T., Bowie, R.C.K., Lafontaine, R.M., Scofield, R.P., Lou, Y., Somarathna, L., Lepage, D., Illif, M., Neuschulz, E.L., Templin, M., Dehling, D.M., Cooper, J.C., Pauwels, O.S.G., Analuddin, K., Fjeldså, J., Seddon, N., Sweet, P.R., DeClerck, F.A.J., Naka, L.N., Brawn, J.D., Aleixo, A., Böhning-Gaese, K., Rahbek, C., Fritz, S.A., Thomas, G.H., Schleuning, M., 2022. AVONET: morphological, ecological and geographical data for all birds. Ecol Lett 25, 581–597. https://doi.org/10.1111/ele.13898
